# Sustained clinical and histopathological remission in a patient with eosinophilic esophagitis and type-2 comorbidities at 18 months after discontinuation of dupilumab

**DOI:** 10.1007/s12328-024-02011-z

**Published:** 2024-07-24

**Authors:** Víctor González-Uribe, Carlos Patricio Acosta Rodríguez-Bueno, Zaira Selene Mojica-González, Andrea Malagón-Liceaga, Martín Roberto Basile-Alvarez

**Affiliations:** 1 Pediatric Allergy and Clinical Immunology Department, Hospital Infantil de Mexico Federico Gomez, Mexico City, Mexico; 2 Faculty of Medicine, Universidad La Salle México, Mexico City, Mexico; 3 Mixed Pediatrics Service, Hospital Infantil de Mexico Federico Gomez, Mexico City, Mexico; 4 InmunoQ, Department of Pathology and Molecular Biology, Mexico City, Mexico; 5 Faculty of Medicine, Universidad Nacional Autonoma de Mexico, Copilco Universidad, Escolar 411A, Coyoacán, 04360 Mexico City, Mexico

**Keywords:** Eosinophilic esophagitis, Type-2 inflammation, Remission, Dupilumab, Biologics

## Abstract

Eosinophilic esophagitis (EoE) is a chronic, allergen-mediated, type-2 inflammatory disease with the potential to significantly impact an individual’s quality of life. Conventional treatments often result in varied responses, prompting the need for novel therapeutic approaches. We present the case of a 19-year-old male with a medical history marked by eosinophilic esophagitis, severe atopic dermatitis (AD), asthma, and allergic rhinitis. Despite undergoing diverse topical and systemic interventions to address his AD and EoE, the patient’s symptoms persisted. However, following the initiation of dupilumab therapy—a dual IL-4 and IL-13 receptor antagonist—the patient experienced a substantial reduction in his Eczema Area and Severity Index score. Notably, a marked improvement was also seen regarding his symptoms of eosinophilic esophagitis. A subsequent esophageal biopsy revealed a significant decrease in eosinophilic inflammation, consistent with established clinical and histologic remission criteria. These findings corroborate the patient’s reported relief from symptoms. This case underscores the potential efficacy of dupilumab as a promising therapeutic agent in managing eosinophilic esophagitis. Dupilumab offers a dual benefit of alleviating symptoms and achieving histologic and clinical remission. This novel approach presents a noteworthy advancement in the treatment of EoE.

## Introduction

Eosinophilic esophagitis (EoE) is a chronic, progressive, cellular-mediated, type-2 inflammatory disease. It is characterized by dysphagia, reflux, and food bolus obstruction in adults, which can lead to esophageal fibrosis or strictures if left untreated and significantly impact quality of life. EoE is diagnosed based on histologic evidence of eosinophilic inflammation in the esophagus, and biopsy is as a key diagnostic tool. A diagnosis of EoE requires a minimum of 15 tissue eosinophils per high-power field (HPF) in esophageal biopsy samples, along with the absence of alternative causative factors and symptom correlation [1, 2].

Histologic definitions of remission of EoE differ, but the most stringent criteria define it using a range of 0 to ≤ 5 eosinophils/HPF [3]. Current treatment options include food elimination diets, proton pump inhibitors, and oral or local glucocorticoids, which demonstrate considerable variability in their response and remission rates [4]. Consequently, there is a need for treatments that specifically target the underlying inflammatory mechanisms and effectively prevent or manage disease progression.

EoE has the potential to impact individuals of all ages, encompassing both children and adults. However, adolescents dealing with EoE encounter distinct clinical considerations. Key among these considerations are dysphagia, food impaction, weight loss, nutritional deficiencies, effects on quality of life, as well as anxiety and emotional distress. The management of EoE in adolescents can pose challenges, given that it frequently involves dietary limitations, medication usage, and consistent check-ins with healthcare professionals. Adherence to treatment and the upkeep of dietary restrictions often prove difficult during adolescence, a period marked by peer influence and social occasions centered around food.

Dupilumab is a monoclonal antibody that was developed to specifically inhibit the IL-4Rα subunit, which is shared by the IL-4 and IL-13 receptors. These interleukins play a crucial role in the underlying mechanisms of type-2 inflammation [5]. Recently, dupilumab received Food and Drug Administration (FDA) approval specifically for treating EoE [6], which marks a significant advancement in addressing this condition. However, further studies will offer valuable insights into optimal dosing and potential adverse effects, which could enhance the utilization of dupilumab for EoE treatment.

One of the main concerns among clinicians is the duration of therapy and its capacity to maintain long-term remission even after treatment discontinuation, particularly for chronic and relapsing conditions like EoE. We present a case illustrating clinical and histopathological resolution in a patient with EoE and type-2 comorbidities. This remission persisted for 18 months after ceasing the use dupilumab following 3 years of biologic therapy. Notably, the patient achieved sustained remission without requiring additional anti-inflammatory drugs or immunosuppressants.

## Case report

A 19-year-old male with a medical history of EoE, severe atopic dermatitis (AD), moderate asthma, and allergic rhinitis presented for evaluation. His family history included allergic rhinitis, asthma, and a father with EoE, who needed esophageal dilations in early adulthood due to stricture formation. The patient had been dealing with EoE symptoms for 5 years, including dysphagia and chest pain related to solid foods, as well as gastroesophageal reflux. Previous evaluations included a complete blood count, which indicated eosinophilia of 5%, total eosinophil count of 450 cells/mm^3^, and total IgE of 113 IU/L. Skin prick tests were performed, which showed positive results for *Fraxinus excelsior* and *Blomia tropicalis.* Antibody evaluations were also performed, which ruled out any other vasculitis or autoimmune diseases.

**Fig. 1:**
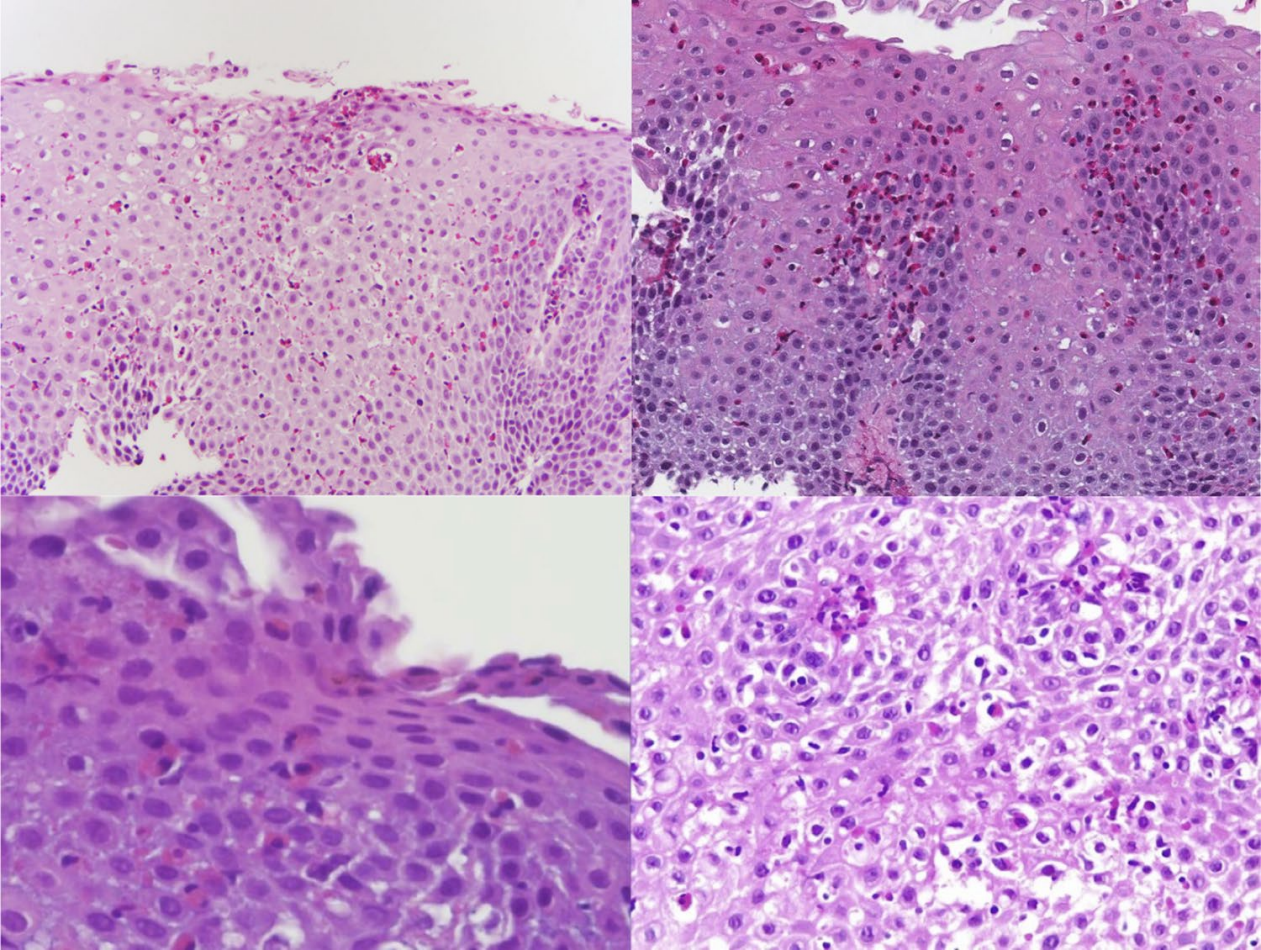
Esophageal biopsies prior to initiating dupilumab treatment showing active esophagitis with ≥ 15 eosinophils/HPF

Previous treatment with omeprazole and oral viscous budesonide provided limited relief of symptoms and histologic improvement. Esophageal biopsies conducted at depths of 21, 24, and 26 cm showed active esophagitis with 15 to 25 eosinophils per HPF and mild to moderate squamous hyperplasia (Fig. 1). Biopsies at the gastric and duodenal levels were also performed, which did not show high eosinophil counts. For this reason, only a diagnosis of EoE was established. The Endoscopic Reference Score (EREFS) at this time of evaluation was 5 points.

**Fig. 2:**
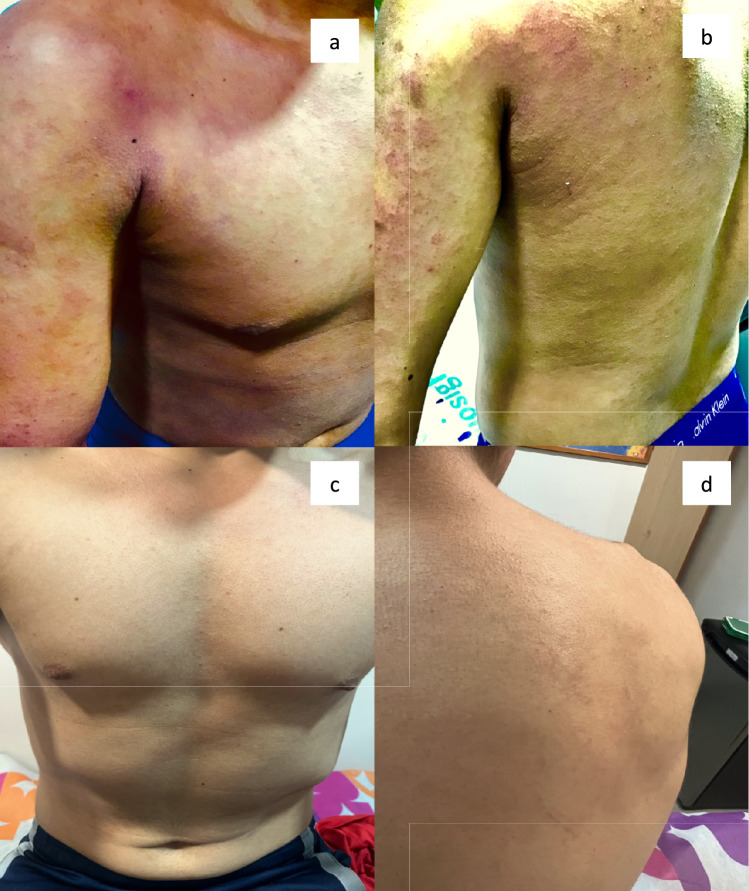
Widespread erythematous and excoriated plaques before dupilumab treatment (**A**, **B**). Significant improvement in dermatological lesions after 18 months of dupilumab drop-off treatment (**C**, **D**)

Despite experiencing numerous gastrointestinal symptoms, the primary reason for the consultation was skin involvement. The physical examination revealed xerosis and extensive, erythematous, excoriated plaques, which predominantly affected the neck, upper back, anterior thorax, antecubital folds, popliteal fossae, and feet (Fig. 2). In previous evaluations, other doctors had used prednisone at 5 mg/day as a treatment for 4 months to try to provide better control of the inflammatory lesions and pruritus due to the extensive skin involvement. The severity of the skin condition was evident with an Eczema Area Severity Index (EASI) score of 34/75, a Patient-Oriented Eczema Measure (POEM) score of 24/28, and an Atopic Dermatitis Control Tool (ADCT) score of 18/24, indicating that the disease was severe, poorly controlled, and burdensome.

Various attempts at topical and systemic treatments were applied for AD, including high-potency topical and oral steroids, topical tacrolimus, cyclosporine, antibiotics, Ultraviolet B (UVB) phototherapy, methotrexate and the current prednisone, but the patient’s symptoms persisted. Consequently, dupilumab therapy was initiated, and the patient’s response to treatment was closely monitored. The patient’s treatment plan involved initiating dupilumab at an initial dose of 600 mg, followed by a maintenance dose of 300 mg every 2 weeks for 2.5 years. As the patient showed satisfactory clinical progress, the maintenance dose was then reduced to 300 mg every month for 6 months. Throughout the treatment period, no significant adverse effects were observed, and the medication was well tolerated.

Within just 5 weeks of treatment, the patient experienced a notable decrease in pruritus, a 75% reduction in EASI (EASI-75), and a substantial improvement in dermatological findings. After tapering the steroid, oral prednisone was also discontinued at this time (Fig. 2). By 24 weeks from the start of treatment, the patient reached a 100% reduction in EASI (EASI-100), demonstrating a substantial resolution of symptoms. Furthermore, there was a remarkable reduction in both POEM and ADCT scores, indicating effective disease control with minimal burden. Currently, the patient’s treatment regimen solely consists of daily application of emollient cream.

**Fig. 3:**
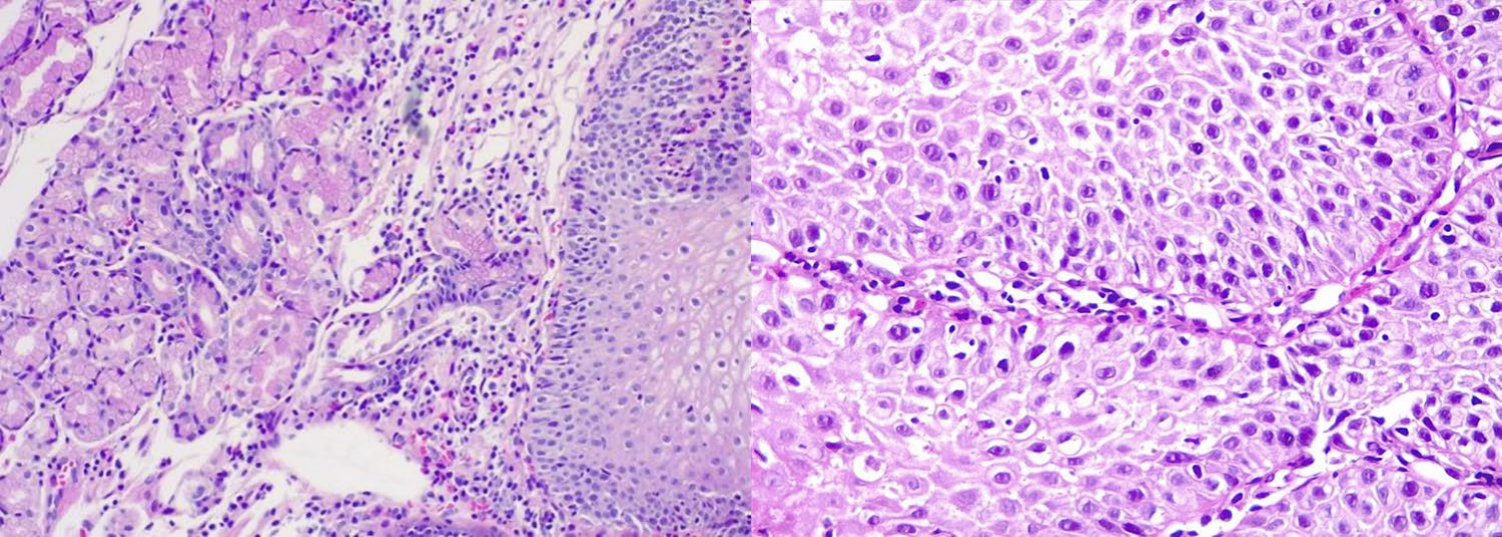
Esophageal biopsies 18 months after dupilumab drop-off treatment with no eosinophils/HPF

**Fig. 4:**
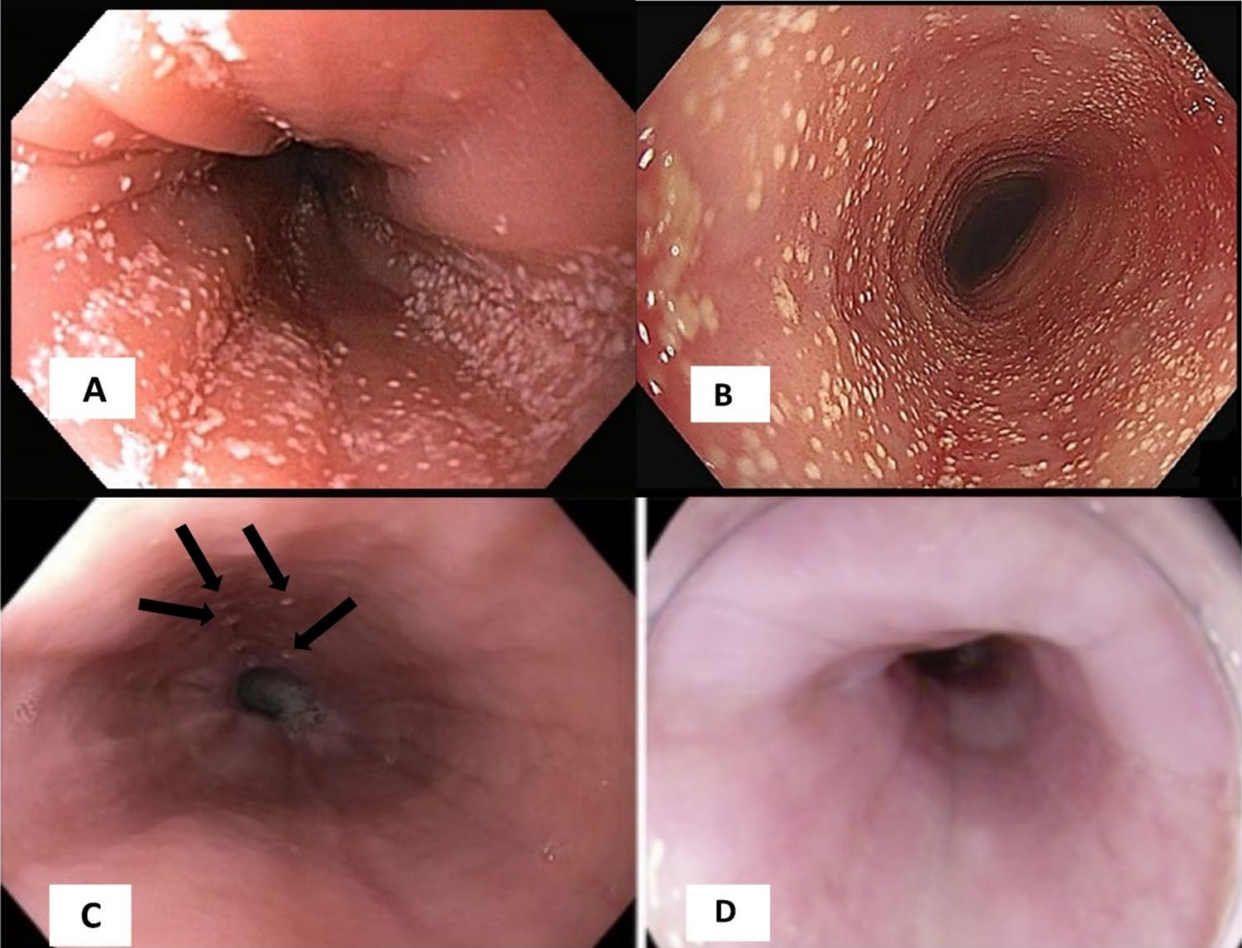
Endoscopic findings. **A** First endoscopy performed at 14 years of age when the patient started having symptoms; **B** Endoscopy performed at 16 years of age after high doses of proton inhibitors and milk-restricted diet, some signs of progression such as trachealization and edema in the esophagus are observed. **C** Exudates, edema, and vascular effacement were observed at the start of treatment with dupilumab showing erythema of the mucosa, longitudinal furrows, vascular effacement, and the presence of microabscesses (arrows), which are consistent with EoE; Patient received oral corticosteroids (5 mg/day of prednisone and oral budesonide). **D** Endoscopy performed 18 months after suspension of dupilumab where no active evidence of the disease

At the 12-week mark of Dupilumab therapy, the patient experienced significant improvement in EoE symptoms, leading to discontinuation of medications such as proton pump inhibitors and oral budesonide. After 24 weeks, he decided to stop limiting problematic foods (dairy and tomato) that had previously triggered symptoms or posed a risk of food impaction, and no adverse clinical effects observed. Follow-up biopsies conducted at 35 weeks indicated only mild reactive changes with ≤ 5 eosinophils per HPF at a depth of 34 cm and rare to absent eosinophils at depths of 28 cm and 31 cm from the esophageal surface, with 1-point EREFS in the endoscopic evaluation. The patient was able to follow a normal diet without restrictions. These findings align with the criteria for remission EoE,

Following completion of the treatment regimen, the patient underwent regular follow-up assessments, revealing significant clinical improvement. At the 18-month milestone after the conclusion of dupilumab treatment, an endoscopic examination was conducted to evaluate the status of the EoE (Fig. 3). The results of the endoscopy showed no abnormalities, indicating an absence of esophagitis and an EREFS score of 0 points in the endoscopic evaluation (Fig. 4). Furthermore, the patient remained asymptomatic with no reported recurrence of dysphagia or gastroesophageal reflux symptoms. Currently, the patient continues to have no need for pharmacologic treatment.

## Discussion

In the present case, the administration of dupilumab demonstrated efficacy as a treatment strategy for EoE and provided both symptomatic relief and histopathological remission. This report contributes significantly to the expanding body of evidence supporting the use of dupilumab in the management of EoE, as observed in recent clinical trials [7]. By effectively inhibiting the inflammatory activity mediated by T2 interleukins, which play a role in the pathogenesis of EoE and other related conditions [8], dupilumab demonstrates its therapeutic potential.

The standard dupilumab regimen for treating EoE typically involves a weekly subcutaneous injection of 300 mg. However, in this case, the patient achieved histopathological remission with a regimen commonly used for managing AD [6]. This suggests that dupilumab remains effective even with less frequent dosing intervals. This finding is particularly relevant because longer intervals between doses may potentially result in fewer adverse effects associated with dosing or long-term administration, including ocular symptoms [10].

This observation introduces an interesting perspective indicating that patients with EoE who also concurrent atopic conditions have, such as severe AD in this case, may potentially derive benefits from early initiation of dupilumab [9]. This approach could facilitate the consolidation of medical treatments, thereby optimizing the overall-management strategy. However, the most notable aspect to emphasize is the sustained remission of the disease even after discontinuing biologic therapy, which has not been previously reported, especially considering the severity and persistence of the disease prior to initiating biologic therapy [11, 12].

Currently, we lack a plausible explanation for the phenomenon observed in this patient, particularly considering that his type-2 diseases are currently asymptomatic. Given that the clearance and elimination of the drug exceed the values reported in pharmacokinetic studies, it is not possible to attribute the effect to pharmacodynamic or pharmacokinetic factors. One hypothesis is that the biologic therapy has induced epigenetic changes in proinflammatory or disease-risk genes, resulting in the suppression of immunoallergic comorbidities. Downregulation of receptors and proinflammatory cytokine levels has been reported in other types of biologic therapies [13, 14], epigenetic changes have also been reported with the application of biologic products as vaccines [15, 16]. Only the upcoming months of follow-up will ascertain whether this remission can endure over the long term for immunologically rooted conditions. In fact, an interesting study with biologic therapy for severe immunology diseases (Rheumatoid Arthritis), shows that at week 12 they maintained their response at week 24 without continuing treatment, indicating a prolonged remission effect of biologics [17]. Nevertheless, this case report establishes a partial precedent in addressing a common query that clinicians encounter — the duration for which biologic therapy will retain its efficacy in such patients. Given the clinical and histopathological evidence, it seems that, for this patient, the answer is at least 3 years.. This suggests that less frequent dosing may be considered for future clinical studies.

This case emphasizes the potential of dupilumab in treating EoE by blocking IL-4 and IL-13, which play a role in the expression of adhesion molecules facilitating eosinophil migration. Sustained clinical and histopathological enhancements underscore the plausibility of a notably more efficacious and secure therapeutic alternative for affected patients. This potential is particularly promising when weighed against the constrained efficacy and concomitant side effects characterizing existing treatment modalities, which often impose a substantial burden on the quality of life experienced by these individuals.

## Abbreviations

AD: Atopic dermatitis
ADCT: Atopic dermatitis control tool
EASI: Eczema area and severity index
EoE: Eosinophilic esophagitis
FDA: Food and drug administration
HPF: High-power field
IL: Interleukin
POEM: Patient oriented eczema measure
UVB: Ultraviolet B
